# miR-183 regulates autophagy and apoptosis in colorectal cancer through targeting of UVRAG

**DOI:** 10.18632/oncotarget.6732

**Published:** 2015-12-22

**Authors:** Longtao Huangfu, Haihai Liang, Guojie Wang, Xiaomin Su, Linqiang Li, Zhimin Du, Meiyu Hu, Yuechao Dong, Xue Bai, Tianyi Liu, Baofeng Yang, Hongli Shan

**Affiliations:** ^1^ Department of Pharmacology State-Province Key Laboratories of Biomedicine-Pharmaceutics of China, Key Laboratory of Cardiovascular Research, Ministry of Education, Harbin Medical University, Harbin, Heilongjiang 150081, P. R. China; ^2^ Department of General Surgery, The First Affiliated Hospital of Harbin Medical University, Harbin, Heilongjiang 150081, P. R. China; ^3^ Institute of Clinical Pharmacy, The 2nd Affiliated Hospital, Harbin Medical University, Harbin, Heilongjiang 150081, P. R. China

**Keywords:** autophagy, UVRAG, miR-183, apoptosis, colorectal cancer

## Abstract

Ultraviolet radiation resistance-associated gene (UVRAG) is a well-known regulator of autophagy by promoting autophagosome formation and maturation. Multiple studies have implicated UVRAG in the pathogenesis of colorectal cancer. However, the mechanisms underlying the regulation of UVRAG are unclear. Here, we describe miR-183 as a new autophagy-inhibiting miRNA. Our results showed that induction of autophagy lead to down-regulation of miR-183 in colorectal cancer cells. And, over-expression of miR-183 resulted in the attenuation of rapamycin- or starvation-induced autophagy in cancer cells, whereas inhibition of endogenous miR-183 stimulated autophagy and apoptosis. Additionally, either autophagy inhibitor 3-MA or pan-caspase inhibitor Z-VAD-FMK respectively or both treatments reversed AMO-183-induced cell death. Further studies showed that UVRAG is a target of miR-183 and as a key regulator promotes autophagy and apoptosis. More importantly, over-expression of UVRAG rescued autophagic activity and induced apoptosis in presence of miR-183. Therefore, the present study investigated the promoting effect of miR-183 on colorectal cancer progression, which was considered to be mediated by autophagy and apoptosis through targeting of UVRAG.

## INTRODUCTION

Autophagy is a highly regulated biological mechanism responsible for intracellular degradation and recycling of proteins and even whole organelles such as mitochondria, and which has been implicated in tumor suppression and anticancer therapy resistance. [1] The ULK1 kinase, which formed an active complex with Beclin-1/PI3K, is believed to master the induction of autophagy. [2] Importantly, the Beclin-1/PI3K complex is a switch between autophagy and apoptosis in the development of cancer, [3, 4] UVRAG (UV radiation resistance-associated gene), a mammalian homolog of yeast Vps38, activated the Beclin-1/PI3K complex, which promoted autophagosome formation. [5] Moreover, UVRAG promoted autophagosome maturation by recruiting HOPS complexes and Rab7 of the late endosome. [6, 7]

Recent studies have demonstrated that autophagic cell death may serve as a novel way to eliminate tumor cells. [8–11] Autophagic proteins that control nucleation and elongation regulate intrinsic apoptosis through Bcl-2 family- and caspase family-mediated cleavage of autophagy-related proteins, which switches the cellular program from autophagy to apoptosis. [12–14] However, it is still needed to further investigate the crucial factors governing the crosstalk between autophagy and apoptosis and to describe the mechanisms controlling cell survival and death. [15]

In recent years, the emergence of microRNAs (miRNAs) as regulators in autophagy has heightened the understanding of the role of autophagy in the pathogenesis of human diseases. [16] To date, numerous miRNAs have been documented to directly regulate autophagic signaling in certain cancer cell lines, including miR-130a (targets ATG2B and DICER1), [17] miR-376b (targets Beclin-1 and ATG4c), [18] miR-181a (targets ATG5) [19]. These findings support the notion that miRNAs regulate autophagy-regulated genes, thereby largely influencing the development of cancer.

miR-183 has been shown to play a potential oncogenic role in multiple cancers. Some studies showed that miR-183 was upregulated in colorectal cancer. [20–23] Here, we describe the role of miR-183 in the control of autophagy. We found that UVRAG, a novel miR-183 target identified, is an important autophagic and apoptotic inducer. Importantly, enhanced expression of UVRAG alleviates miR-183-mediated repression of autophagy and induces apoptosis in colorectal cancer cells, confirming the functional importance of this target.

## RESULTS

### Down-regulation of miR-183 in Rapamycin- or Starvation-induced autophagy in HCT116 and HT29 cells

**Figure 1:**
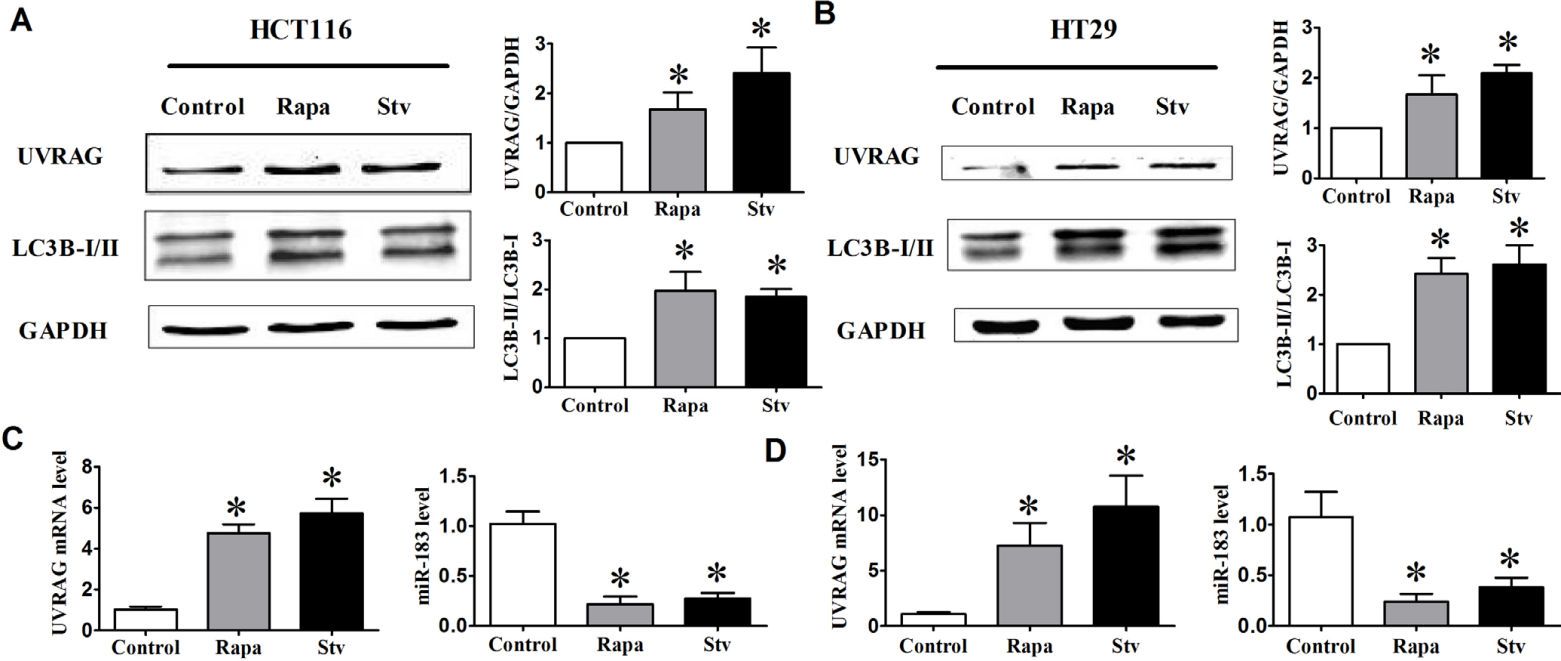
The expression of UVRAG and miR-183 after autophagy induction HCT116 and HT29 cells were treated with rapamycin (1 μM, 12 h) or incubated in HBSS for 4 h (starvation condition). (**A**) and (**B**) Autophagy activation was monitored based on the LC3B-I to LC3B-II conversion and the UVRAG expression. The relative expression level of UVRAG and miR-183 in HCT116 cell (**C**) or HT29 cell (**D**) was assessed by qPCR. *n* = 4. *p < 0.05 vs control.

Recent reports showed several genome-wide microRNA profiling studies to find autophagy-relevant microRNAs. [24–26] Among the miRNAs identified to repress autophagy, miR-183 was immediately interesting due to well-established links to colorectal cancer. To explore the possible links between autophagy and miR- 183 expression, we measured the level of endogenous miR-183 under basal growth conditions and following induction of autophagy. As shown in Figure 1A and 1B, starvation condition or rapamycin treatment triggered a distinct increase of LC3B-II/LC3B-I ratio. Meanwhile, quantitative RT-PCR (qRT-PCR) analysis revealed that endogenous miR-183 expression is decreased by starvation or rapamycin treatment. In addition, both UVRAG protein level and mRNA level were increased following induction of autophagy (Figure 1A–1D). These data indicated a potential physiological role for endogenous miR-183 in autophagy regulation in colorectal cancer cells, prompting us to further analyze its function.

### Role of endogenous miR-183 in the control of autophagic activity

**Figure 2:**
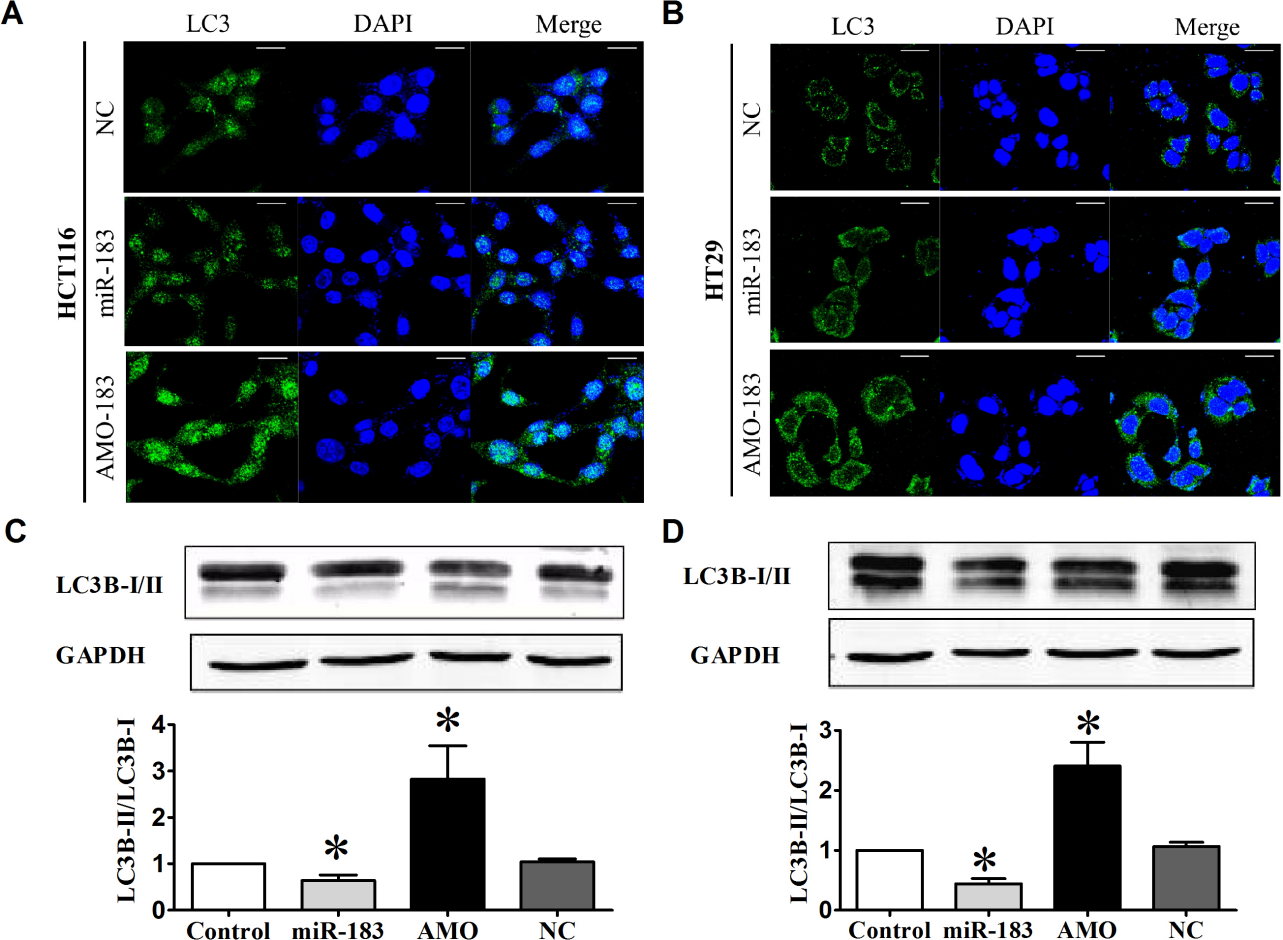
miR-183 affected autophagic activity in colon cancer cells HCT116 and HT29 cells were transfected with miR-183 mimic or AMO (100 nM). (**A**) and (**B**) LC3 puncta were visualized by confocal imaging 48 h after transfection. Scale bars represent 20 μm. (**C**) and (**D**) LC3B-I to LC3B-II conversion were detected by Western Blot. *n* = 4. *p < 0.05 vs control.

To validate the repressive effect of miR-183 on autophagy, we transfected miR-183 mimics into HCT116 cells and HT29 cells. Our data showed that the number of LC3 puncta was significantly reduced in cells overexpressing miR-183 (Figure 2A and 2B). Next, we assayed the effect of miR-183 on autophagy. In line with previous resulted, over-expression of miR-183 both in HCT116 and HT29 cells resulted in decreased LC3B-II/LC3B-I ratios (Figure 2C and 2D). These results introduced miR-183 as a new inhibitor of autophagy. To further test the effects of miR-183 inhibition on autophagy, we transfected cells with miR-183 specific inhibitor (AMO-183) and analyzed autophagic activity. As expected, the LC3 dots formation were increased (Figure 2A and 2B) and LC3B-I to LC3B-II conversion were stimulated (Figure 2C and 2D) both in HCT116 and HT29 cells following the introduction of AMO-183. These results indicated that endogenous miR- 183 contributes to the limitation of autophagic responses in colorectal cancer cells.

### Inhibition of miR-183 promotes cell death in HCT116 and HT29cells

**Figure 3:**
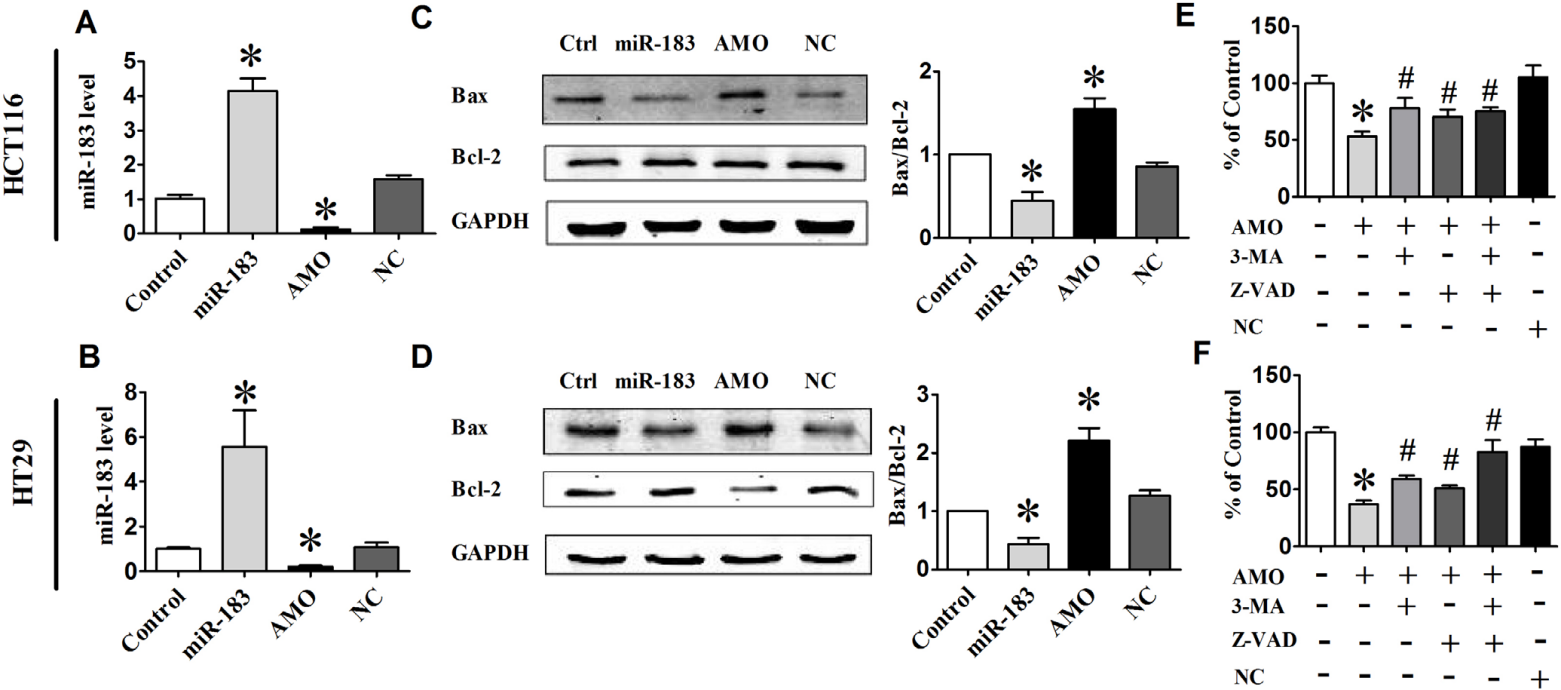
miR-183 regulated cell death in colon cancer cells (**A**) and (**B**) qPCR analysis of miR-183 expression levels in HCT116 and HT29 cells following transfected with miR-183 or AMO-183. (**C**) and (**D**) The expression levels of Bax and Bcl-2 analyzed by Western Blot 48 h after transfection. (**E**) and (**F**) Cell viability was determined by MTT assay. 3-MA, an autophagy inhibitor. Z-VAD-FMK, an apoptosis inhibitor. *n* = 3. *p < 0.05 vs control; #*p* < 0.05 vs AMO.

In an effort to explore the possible role of miR-183 in cell death, we transfected HCT116 and HT29 cells with miR-183 or AMO-183. qRT-PCR analysis showed that the miR-183 level was approximately 5-fold higher in miR-183 transfected group compared with the control group, and AMO-183 effectively inhibited the expression of miR- 183 (Figure 3A and 3B). Inhibition of endogenous miR-183 caused a significant increase of the ratio of Bax/Bcl-2 in HCT116 and HT29 cells (Figure 3C and 3D). Furthermore, cell viability was also decreased in AMO- 183-transfected cells indicating that miR-183 plays an important role in the regulation of cell death. Importantly, the effect of AMO-183 was reversed upon treatment of either 3-MA [27] or pan-caspase inhibitor Z-VAD-FMK [28] respectively or both (Figure 3E and 3F). These results suggest that AMO-183 induces cell death through both apoptosis- and autophagy-dependent signaling pathway.

### Experimental identification of autophagy-related target of miR-183

**Figure 4:**
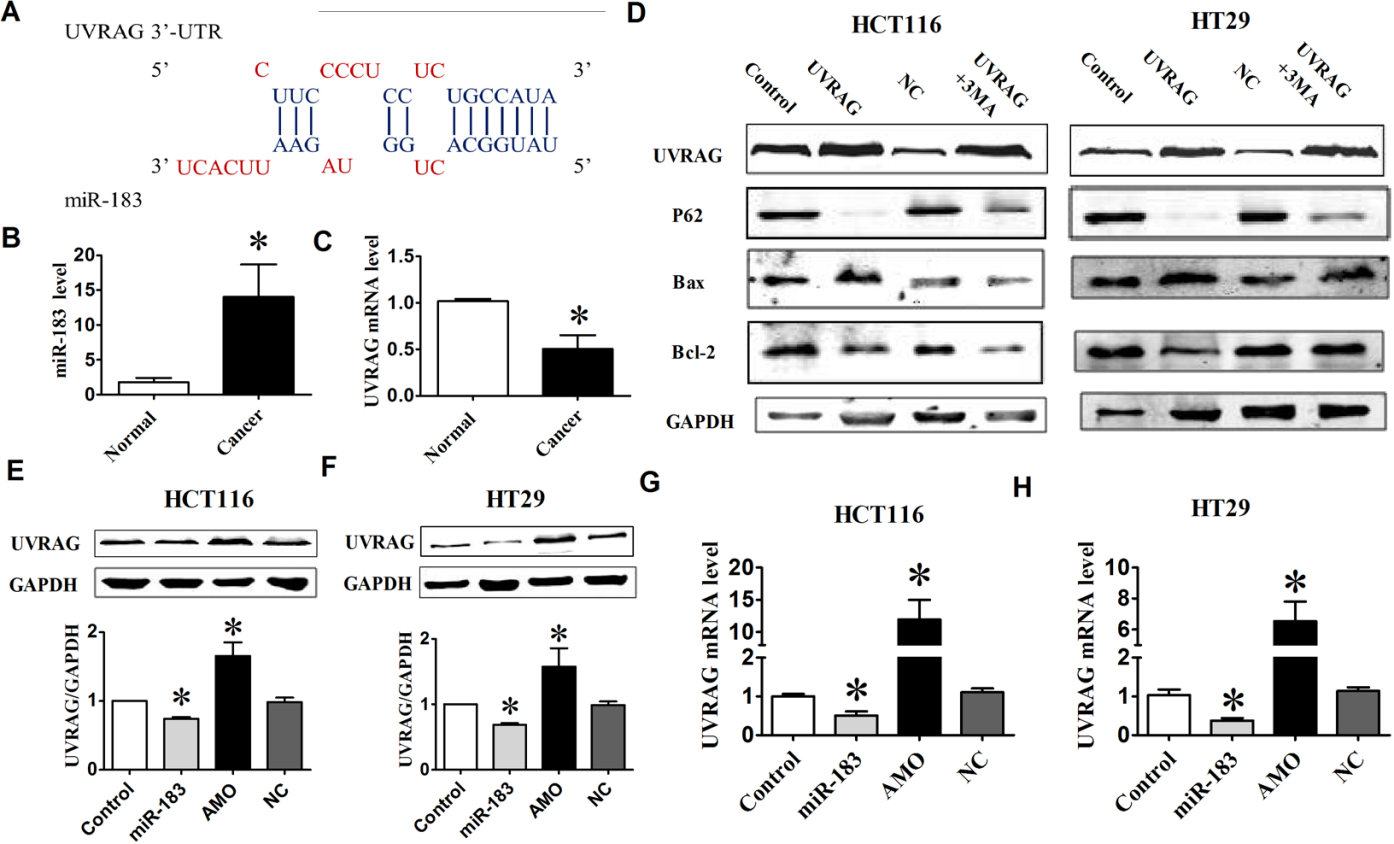
miR-183 affected UVRAG expression levels (**A**) miR-183 mature miRNA sequence and miR-183 binding target sequences in the 3′-UTR of UVRAG. (**B**) The UVRAG mRNA level was determined by qPCR in 7 paired human colorectal cancer and adjacent normal tissues. (**C**) Analysis of the level of miR-183 in colorectal cancer tissues. (**D**) Western Blot analysis of UVRAG, P62, Bax, Bcl-2 in HCT116 or HT29 cells under over-expression of UVRAG. (**E**) and (**F**) HCT116 and HT29 cells were transfected with miR-183 or AMO-183, respectively, and UVRAG protein levels were detected by Western Blot. (**G**) and (**H**) qPCR analysis of UVRAG mRNA and miR-183 expression levels in HCT116 and HT29 cells following transfection of miR-183 or AMO-183. *n* = 4. *p < 0.05 vs control.

Having established the role of miR-183 on autophagy, we wanted to clarify the underlying mechanism by identifying the direct downstream targets. We searched for autophagy genes containing potential miR-183 MREs in their 3′UTRs using publicly available bioinformatics tool DIANA-microT-v4. And, UVRAG was identified as a miR-183 target by microT-v4 (Figure 4A). Indeed, most tumor tissues from colorectal cancer patients showed higher levels of miR-183 compared with the corresponding normal tissues (Figure 4B). In contrast, the level of UVRAG mRNA was significantly lower in tumor tissues than that in normal tissues (Figure 4C). These data suggested that UVRAG might be involved in the regulation of miR-183 during colorectal cancer development. Further studies showed that over-expression of UVRAG significantly increased the ratio of Bax/Bcl- 2 and the degradation of p62, suggesting that UVRAG promotes apoptosis and autophagy in colon cancer cells, and treatment of cells with 3-MA inhibited UVRAG-induced apoptosis and autophagy (Figure 4D).

To confirm the bioinformatics-based predictions, we performed Western Blot analysis of UVRAG expression in miR-183 or AMO-183 transfected cell. As shown in Figure 4E and 4F, over-expression of miR-183 inhibited UVRAG at protein levels in HCT116 and HT29 cells. Conversely, inhibition of endogenous miR-183 resulted in an increase in the expression of UVRAG compared with control group. Moreover, a decrease in UVRAG mRNA levels was observed in cells following transfection with miR-183, and was reversed by introducing AMO-183 (Figure 4G and 4H).

### miR-183 antagonizes rapamycin- and starvation-induced cell death in colorectal cancer cells

**Figure 5:**
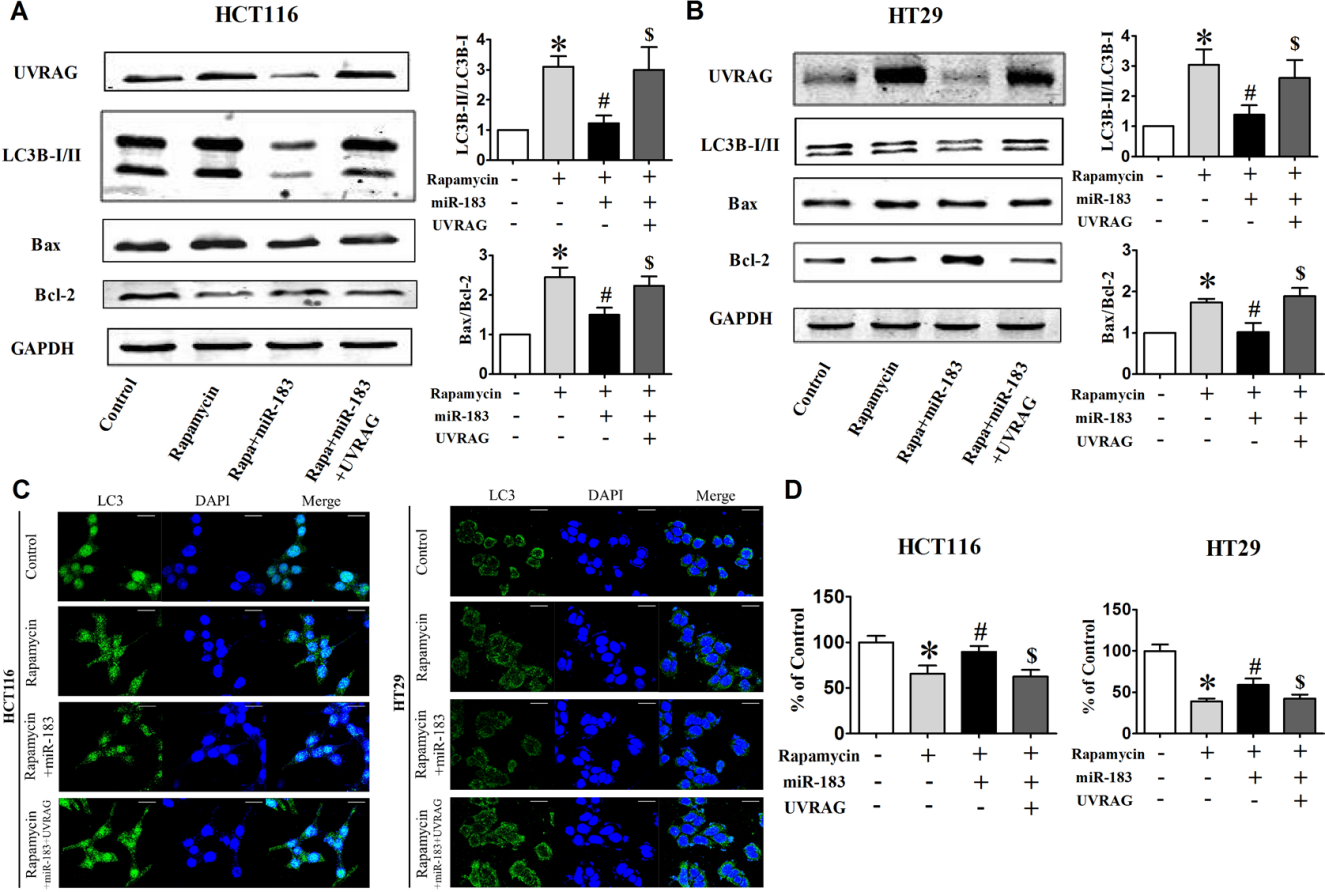
Over-expression of UVRAG rescued cells from miR-183-mediated autophagy inhibition HCT116 and HT29 cells were cotransfected with miR-183 and UVRAG expression plasmid lacking the miR-183 target region. (**A**) and (**B**) Western Blot analysis of UVRAG, LC3-I/II, Bax, Bcl-2 in HCT116 and HT29 cells after cotransfection. (**C**) LC3 puncta were visualized by confocal imaging before or after rapamycin treatment (12 h). Scale bars represent 20 μm. The LC3 puncta were quantitated by randomly counting 10 cells for each group. (**D**) Cell viability was determined by MTT assay. *p < 0.05 vs control; #*p* < 0.05 vs rapamycin; $*p* < 0.05 vs rapamycin + miR-183.

It is well established that various chemotherapeutics induce autophagy in different cancer cells including colorectal cancer cells. Rapamycin has also been recognized as potent immunosuppressive and antitumor agent. [29] Studies in a variety of human cell lines have linked the antitumor activity of rapamycin to the induction of either G1 arrest (cytostasis) or apoptotic death. We turned to rapamycin treatment to further establish a functional importance of miR-183 in autophagy-related cellular phenotypes. The LC3B-I to LC3B-II conversion (Figure 5A and 5B) and LC3 dots formation (Figure 5C) were suppressed by miR-183 during rapamycin-induced autophagy, which was reversed upon co-expression of the UVRAG. We then examined the ability of miR-183 to reduce rapamycin-mediated cell death. The combinatorial treatment of rapamycin and miR-183 significantly enhanced cellular survival and desensitized cells to rapamycin-induced apoptosis as evidenced from decreased Bax/Bcl-2 ratio (Figure 5A and 5B) and cell viability (Figure 5D). However, the miR-183-mediated survival response observed during rapamycin-induced cell death was reversed upon co-expression of UVRAG.

**Figure 6:**
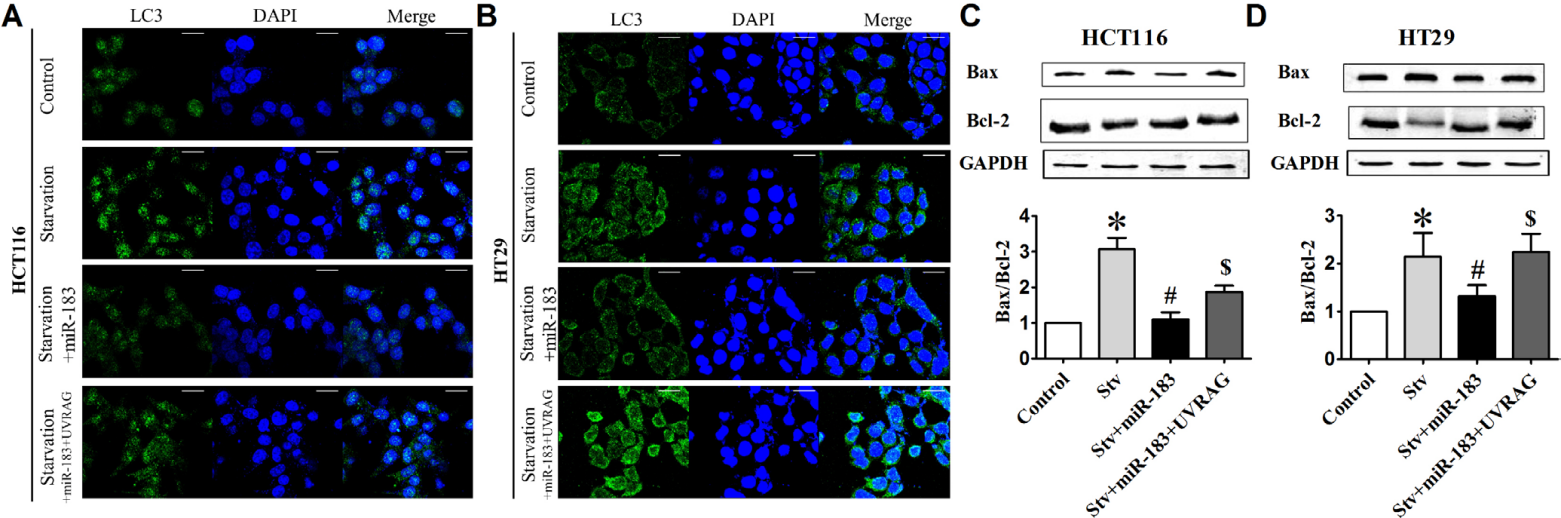
miR-183 antagonized starvation-induced cell death in colon cancer cells (**A**) HCT116 and (**B**) HT29 cells were transfected with miR-183 and LC3 puncta were visualized by confocal imaging after starvation. Scale bars represent 20 μm. The GFP dots were quantitated by randomly counting 10 cells for each group. (**C**) and (**D**) Western Blot showed Bax and Bcl-2 expression levels in HCT116 and HT29 cells after miR-183 transfection. *n* = 3. *p < 0.05 vs control.

Similar to the rapamycin-related result, starvation-induced LC3 dots formation (Figure 6A and 6B) and Bax/Bcl-2 ratio enhancement (Figure 6C and 6D) was also reversed upon transfected with miR-183 both in HCT116 and HT29 cells. These results indicated that UVRAG is the functional element in miR-183-mediated anti-apoptosis response.

### Down-regulation of miR-183 suppresses colon tumor growth *in vivo*

**Figure 7:**
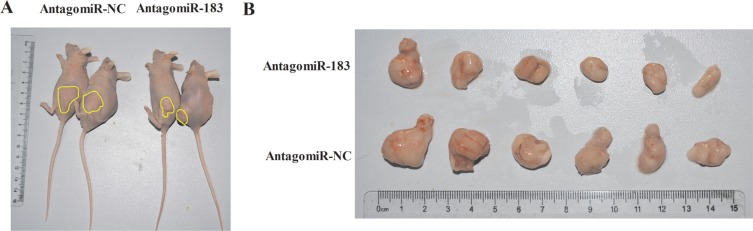
AntagomiR-183 inhibited colon tumor growth *in vivo* 1 × 10^6^ HT29 cells were subcutaneously injected into Nu/Nu mice. After the tumor was established, tumors were directly injected with 4 nmol antagomiR-183 in 50 μl PBS followed by measuring of tumor size until 2 weeks when mice were sacrificed. (**A**) Representative images of mice bearing HT29 tumors and (**B**) gross morphology of tumors after dissection were shown.

To extend our observations from cultured cells, we further investigated the role of miR-183 in mouse colon cancer xenografts models. Intratumoral injections of antagomiR-183 significantly reduced the volumes of subcutaneous tumors (Figure 7), indicating that antagomiR-183 may be a novel adjuvant therapeutic agent for future therapeutic development.

## DISCUSSION

There have been several microRNA studies related to clinical colorectal cancer and most have shown that aberrant expression of miRNAs occurs in colorectal cancer, some of which function as tumor-suppressor genes or oncogenes. [30] Based on the literatures, the level of miR-183 expression in colorectal cancer has previously been described to be higher than adjacent normal tissues, suggesting that miR-183 could be considered to be a promising biomarker for early colorectal cancer detection and accurate prognosis as well as targets for more efficient treatment. [31] Indeed, miR-183 has been suggested to be an oncogene in several cancers such as colorectal, lung and hepatocellular, where it regulates diverse mediators of tumor survival and function, including targeting the tumor suppressor Bmi-1, EGR1, PTEN and SMAD4. [32–34] A previous study showed that miR-183 knockdown in medullary thyroid carcinoma cells reduced cellular proliferation in association with elevated LC3B expression. [31] This is suggestive of increased autophagic flux and potential cell death via autophagy induction. Thus, miR-183 may act as a double-edged sword by inhibiting both apoptosis and autophagy in tumor cells.

Colorectal cancer is the second most common cancer in females and the third in males with 1.2 million annual new cases and 600,000 deaths worldwide. Approximately two-thirds will be treated surgically with curative intent. Among those treated curatively, around one-third will experience recurrence of the original cancer or a second primary (i.e., metachronous) colorectal cancer. [35] Generally, patients were applied chemotherapeutic drugs to prevent tumor recurrence after surgery. Chemotherapy suppresses cancer development through inducing apoptosis and inhibiting cell cycle. [36] However, cancer is a complex disorder associated with defects in multiple signaling pathways that confer resistance to apoptosis.

It has been shown that impaired autophagy is intimately associated with the pathogenesis of many human diseases. For example, decreased expression of Beclin-1 is associated with the development or progression of human malignancies. Autophagy is a complex signaling process involving over 35 autophagy-related gene products and numerous autophagy regulating molecules that act in concert. [37, 38] UVRAG is required for autophagy through the involvement of Beclin-1-PI3K C3 complex in the formation of autophagosomes. [39] Autophagy may represent an important mechanism in the interaction of genetic susceptibility and metabolic dysregulation that contribute to the pathogenesis of colorectal cancer. [40] Therefore, exploring how UVRAG and autophagy pathways are regulated may increase our understanding of the pathogenesis of colorectal cancer.

Recent studies demonstrated that UVRAG not only promotes Beclin-1-mediated autophagy, but it also facilitates endosome/autophagosome maturation. [7, 39, 41] Zhao Z *et al*. found that genotoxic and metabolic stress increase the expression of UVRAG and lead to apoptosis in human tumor cells. [42] Furthermore, UVRAG has drawn special attention recently due to the identification of UVRAG as a risk gene for colorectal cancer. [43–45] Chromosomal 11q deletion involving UVRAG is frequently identified in human colorectal cancer, and is shown to be highly associated with survival probability. [46–48] In this study, we report here that UVRAG promotes autophagy and apoptosis in human colorectal cancer cells, indicating that UVRAG function as autophagy-related tumor suppressor.

In line with previous studies, we confirmed that that miR-183 can affect apoptosis- and autophagy-mediated cell death in colorectal cancer cells through targeting UVRAG. Although UVRAG-mediated autophagy through interaction with Beclin-1 has been documented in mammals, the expression and function of UVRAG during apoptosis may be more complex. Interestingly, one recent study reported that UVRAG binds to Bax in the cytosol and prevents Bax translocation to mitochondria. [49] UVRAG contains an amino-terminal proline-rich sequence followed by a potential calcium-dependent phospholipid binding C2 domain. [50] These evidences indicated that UVRAG not only binds to Bax, but may also interact with other Bcl-2 family members and many inducers of autophagy to cause cell death. In the following research, we will focus on establishing the evidence for the direct interaction between UVRAG and other Bcl-2 family members or BH3-only proteins, and attempt to provide answers by explaining how the change in UVRAG expression is able to affect apoptosis.

In summary, our present study provides evidence for an important role of miR-183 in colorectal cancer for regulating UVRAG-mediated autophagy and apoptosis. Inhibition of miR-183 might provide a potential novel therapeutic strategy for patients with colorectal cancer harboring a constitutively activated autophagy signaling pathway.

## MATERIALS AND METHODS

### Ethics statement

Investigation has been conducted in accordance with the ethical standards and according to the Declaration of Helsinki and according to national and international guidelines and has been approved by the local institutional review board.

### Samples acquisition

A total of 14 frozen colorectal patient specimens were selected (7 paired colon normal mucosa and tumor samples). These patients had undergone surgical resection of primary colorectal adenocarcinoma at the First Affiliated Hospital of Harbin Medical University under the procedures approved by the Ethnic Committee for Use of Human Samples of Harbin Medical University (Harbin, China).

### Animal experiments

All animal experiments were performed in accordance with the NIH Guide for the Care and Use of Laboratory Animals. We performed all animal surgery under ketamine anesthesia, and took every effort to minimize animal suffering. Athymic nude mice (6 weeks old; 20–30 g) were provided by Charles River Laboratories, Inc. The mice were randomly divided into two groups (antagomiR negative control group, *n* = 6; antagomiR-183 group, *n* = 6). HT29 cells (1 × 10^6^) in PBS were inoculated subcutaneously into the flanks of nude mice. Tumor size was measured as described previously. 4nmol antagomiR-183 in 50 μl PBS was directly injected into each tumor. The injection began when tumor volume reached 100–150 mm. Mice were sacrificed after 2 weeks.

### Cell culture and treatment

HCT116 and HT29 cells were obtained from American Type Culture Collection and grown in RPMI 1640 medium (Biological Industries, Kibbutz BeitHaemek, Israel), supplemented with 10% fetal bovine serum (Biological Industries) and antibiotics at 37°C in a 5% CO_2_ incubator. Cells were split regularly and were used between passages 5 and 20 in this study. For autophagy induction by rapamycin (Sigma-Aldrich), cells were incubated in fresh complete medium containing 1 μM rapamycin for 24 h. For autophagy induction by starvation, cells were rinsed twice and then incubated in Hank's Balanced Salt solution (HBSS; Cellgro) for 4 h.

### Cell viability assay

MTT assay was used to determine cell viability. Colorectal cancer cells were washed and replaced with fresh medium. Then the cells were treated as designated and were subsequently incubated with 20 μl MTT (0.5 mg/ml) for 4 hours. The culture medium was carefully removed, 150 μl DMSO was added to each well to dissolve the formazan. After rocking for 10 minutes, the absorbance values were read at 570 nm using an Infinite^®^200PRO microplate spectrophotometer (Tecan, Salzburg, Austria).

### Transfection of miRNAs and plasmids

miR-183 mimics, miR-183 specific inhibitor (miR-183 antisense oligodeoxyribonucleotide, AMO-183), antagomiR and negative control miRNA (NC) were obtained from Genepharma (Shanghai, China). p3xFLAG-CMV-10 plasmid that encodes UVRAG CDS region was generously provided by Dr. Noboru Mizushima (Addgene plasmid, 24292). And the p3xFLAG-CMV-10 plasmid as control vector was obtained from Sigma-Aldrich. Colorectal cancer cells were transfected with 1.0 μg plasmid and 100 nM microRNA respectively or both using X-treme GENE HP^®^ (Roche Molecular Biochemicals, Basel, Switzerland) at the confluence of 70%–80% according to the manufacturer's instruction. The cells were then subjected to different treatment as designated after 36 hours of transfection.

### Confocal imaging

Cells grown on cover glasses were treated as designated. Cells were fixed in 4% paraformaldehyde and permeabilized by 0.5% Triton X-100. Following blocking with 3% bovine serum albumin, cells were serially incubated in rabbit anti-LC3B (Sigma-Aldrich) and Goat anti-rabbit Alexa Fluo488 (Invitrogen). Finally, cells were rinsed and mounted on cover glasses with Prolong Gold anti-fade reagent with 4′-6-diamidino-2-phenylindole (Invitrogen) and the immunostaining was observed under the AV300-ASW confocal microscope (Olympus America Inc., Center Valley, USA) with a 60 × oil lens. The number of LC3 puncta per cell was quantified manually. At least 20 cells in 3 independent experiments were analyzed randomly.

### Western blot

After the treatment described above, cells were lysed in RIPA buffer supplemented with complete protease inhibitor cocktail (Roche Molecular Biochemicals, Basel, Switzerland). The protein concentration was determined by BCA kit (Beyotime, Shanghai, China) according to the instruction. For western blot analysis, 30–60 μg denatured protein were separated in 15% or 12% SDS-polyacrylamide gels and transferred to nitrocellulose membranes. Prior to incubating with primary antibody, the membranes were blocked with 5% skimmed milk in PBS for 2 hour in room temperature on a rocker. The membranes were incubated with diluted antibody in PBS at 4°C, overnight. The membranes were washed 3 times with PBST (PBS containing 0.5% Tween 20) for 10 minutes each, followed with re-probing with fluorescence conjugated secondary antibodies in room temperature for 1 hour. Membranes were washed again for 3 times with PBST for 10 minutes each prior to detections on Odyssey infrared scanning system (LI-COR Biosciences, Lincoln, USA). The Western Blot bands were quantified using Odyssey 3.0 software and normalized with respect to loading control. The antibody resources are as follows:

Antibody against LC3B was obtained from Sigma-Aldrich. Antibody against UVRAG was obtained from Millipore. Antibodies against Bax and Bcl-2 were obtained from Sangon Biotech. Antibody against GAPDH was obtained from Proteintech. The fluorescence conjugated secondary antibodies IRDye700/800 Mouse or Rabbit were obtained from LICOR.

### RNA extraction and quantitative real-time RT-PCR analysis

Total RNA was isolated from cultured cells using a Trizol standard protocol (Invitrogen, Carlsbad, USA). The integrity, quantity, and purity of RNA were examined using Nano-Drop 8000 Spectrophotometer (Thermo Scientific, Wilmington, USA). For each sample, 500 ng of total RNA was converted to cDNA using High Capacity cDNA Reverse Transcripition Kit (Applied Biosystems, Foster City, USA). The relative expression levels of mRNAs and miRNAs were quantified by the mirVanaqRT-PCR miRNA Detection Kit in conjunction with real-time RT-PCR with SYBR Green I (Applied Biosystems). After circle reaction, the threshold cycle (Ct) was determined and relative mRNA and miRNA levels were calculated based on the Ct values and normalized to GAPDH or U6 level in each sample.

### Statistical analysis

All the data are expressed as the mean ± SEM. GraphPad Prism 5.0 was used for the statistical analysis. For 2 treatment groups, the Student *t* test was used. For 3 or more treatment groups, one-way ANOVA was used with Bonferroni post-test for the comparison of selected 2 treatment groups as well as Dunnett post-test for comparing all other treatment groups to the corresponding control. A value *p* < 0.05 was considered statistically significant.

## Abbreviations

UVRAG: ultraviolet radiation resistance-associated gene
ATG: autophagy related genes
miRNA: microRNA
miR-183: human microRNA-183
AMO-183: miR-183 antisense oligodeoxyribonucleotide
mTOR: mechanistic target of rapamycin
LC3B: microtubule-associated protein 1 light chain 3B
Bcl-2: B-cell lymphoma 2
Bax: Bcl-2 associated X protein
RAPA: rapamycin
STV: starvation
qRT-PCR: quantitative RT-PCR;
MTT: thiazolyl blue tetrazolium bromide
HBSS: Hank's Balanced Salt Solution
3-MA: 3-methyladenine
MRE: miRNA-response element
U6: U6 small nuclear 1 (RNU6–1)
GAPDH: glyceraldehyde-3-phosphate dehydrogenase
DAPI: 4′,6-diamidino-2-phenylindole
DMSO: dimethyl sulfoxide
